# Reclassifying Papillary, Oncocytic and Chromophobe Renal Tumours Based on the 5th Who Classification 2022

**DOI:** 10.5146/tjpath.2024.13052

**Published:** 2024-05-18

**Authors:** Nilofar Shaikh, Mary Mathew

**Affiliations:** Department of Pathology, Kasturba Medical College, Manipal, Manipal Academy of Higher Education, Karnataka, India

**Keywords:** Classification, Chromophobe renal cell carcinoma, Oncocytoma, Papillary renal cell carcinoma

## Abstract

***Objective: *** The classification of renal tumors is expanding with the addition of new molecular entities in the 5th World Health Organization classification. Apart from this, the major updates in the definition of papillary renal cell carcinoma are that these tumors are no longer subtyped into type 1 and type 2. In oncocytic tumors, the new molecularly defined renal tumors, emerging and novel entities need to be considered in the diagnosis of oncocytic and chromophobe renal tumors.

***Material and Methods:*** This is a retrospective study to review and reclassify papillary, oncocytic, and chromophobe renal tumors based on the new WHO classification and correlate with clinical data, gross, microscopic features, and immunohistochemistry markers.

***Results: *** A total of thirteen cases were reviewed and the tumor grade was changed for three out of four cases of papillary renal cell carcinoma and a single case was recategorized and graded. In nine cases of oncocytic and chromophobe renal tumors, the diagnoses were modified in 3 cases.

***Conclusion:*** Newly defined molecular renal tumors require advanced immunohistochemistry markers and molecular tests. This poses diagnostic challenges to pathologists practicing in low resource settings where molecular tests are not available.

## INTRODUCTION

Renal tumours account for a high proportion of the morbidity and mortality of cancer deaths. Globally, these tumours are the 14th most common cancers and constitute 2% of all cancers (1,2). The incidence of renal cell carcinoma is comparatively lower in Asian countries, due to under-reporting and scarcity of data. The National Cancer Registry Programme 2012-2016 India has projected an increasing trend in the incidence of renal tumours in 2025 (3).

In 1996, the University of Heidelberg in Germany held a meeting of experts and proposed a new classification, the Heidelberg classification, based on the morphological, clinical, and molecular characteristics of renal neoplasms. This was followed by a consensus conference by the Mayo Clinic, the American Cancer Society, Union Internationale Centre le Cancer (UICC), and American Joint Committee on Cancer (AJCC) leading to the current classification which was adopted by the World Health Organisation (WHO) (1).

Since then, the classification of renal tumours is expanding with more additions of molecular entities, making the diagnosis difficult and challenging but necessary for therapy. The previous 4th edition WHO classification of adult renal tumours was based on the International Society of Urological Pathology (ISUP) Vancouver classification. This taxonomy has been changed in the current edition with the addition of newer entities. The 5th edition WHO classification of urinary and male genital tumours has made significant changes especially in the reclassification of renal tumours based on morphology and molecular studies (4). Molecular diagnostics and immunohistochemistry (IHC) aid in categorising these tumours for targeted therapy. However, many institutions in India lack these facilities to diagnose these tumours. In this study, we attempted to review and reclassify wherever necessary, retrospectively all papillary, oncocytic, and chromophobe tumours diagnosed in the last year based on the new classification, diagnostic criteria, and immunohistochemical markers.

### Recent Major Updates in Renal Tumours

A major addition in the new 5th WHO classification is the inclusion of benign tumours in this classification which was not mentioned in the previous edition. In the current classification, renal tumours are classified into six major groups - Clear cell renal tumours, Papillary renal tumours, Oncocytic and Chromophobe renal tumours, Collecting duct tumours, Other renal tumours and Molecularly defined renal carcinomas. In the categories of Clear cell renal tumours, Oncocytic and Chromophobe renal tumours and Collecting duct tumours, there are no major updates. The new major updates are:

Papillary renal cell carcinoma is no longer subclassified into types 1 and 2. The former Type 1 papillary Renal cell carcinoma is now termed as the classic morphology of papillary RCC. The features of type 2 papillary renal cell carcinoma is now associated with other tumour types like fumarate hydratase deficient RCC, eosinophilic solid cystic RCC, and translocation RCC.

The nomenclature of Clear cell papillary renal cell carcinoma has been changed from ‘carcinoma’ to ‘tumour’ due to its more indolent clinical behaviour.

In Chromophobe renal cell carcinoma, nonconventional morphologies like the trabecular, alveolar, papillary, microcystic or cystic architecture maintain CK7/CD117 co-expression and are associated with favourable prognosis (3).

Previously defined MiT family translocation RCC harboured gene fusions of two genes of the MiT family of transcription factors named TFE3 and TFEB. As per the recent WHO classification, TFE3 rearranged RCC and TFEB altered RCC are now considered as two separate molecularly defined renal cell carcinomas.

The recent updates in the classification of renal tumours are listed in Table 1.

**Table 1:** Recent WHO updates in 5th edition of classification of renal tumors

**Molecularly Defined Renal Carcinomas** i. TFE3-rearranged renal cell carcinoma ii. TFEB-altered renal cell carcinoma iii. ELOC (formerly TCEB1) - mutated renal cell carcinoma iv. Fumarate hydratase-deficient renal cell carcinoma v. Succinate dehydrogenase-deficient renal cell carcinoma vi. ALK-rearranged renal cell carcinoma vii. SMARCB1 deficient Medullary carcinoma	**Novel entities** i. Eosinophilic solid and cystic renal cell carcinoma
	**Emerging Entities** i. Thyroid like follicular carcinoma ii. Other oncocytic tumours – a. Hybrid oncocytic chromophobe tumour (HOCT) b. Eosinophilic vacuolated tumour (EVT) c. Low-grade oncocytic tumour (LOT) iii. Biphasic hyalinizing psammomatous RCC iv. Papillary renal neoplasm with reversed polarity

In addition to this, the 5th edition WHO classification of urinary and male genital tumours has specified diagnostic recommendations for the following tumours: multilocular cystic neoplasm of low malignant potential, Clear cell papillary renal cell tumour and Oncocytoma. These tumours should not be diagnosed on needle biopsies as they can have overlapping features with their malignant counterparts.

## MATERIAL and METHODS

This was a retrospective observational study conducted from January 2020 till May 2023 at the Department of Pathology. Previously diagnosed papillary, oncocytic, and chromophobe renal tumours of the kidney were reviewed and reclassified when required based on the new 5th WHO classification of renal tumours. A total of 13 renal tumours that satisfied the criteria were included in the study. All other renal tumours were excluded. The clinical details and patient demographic data were obtained from the medical records. The slides were retrieved from the archives from the department and the gross, microscopic features and immunohistochemistry (IHC) were reviewed. The panel of IHC markers that were used were CK7, CD117, CD10, AMACR, S100, Vimentin, HMB45, E-cadherin, TTF1 and PAX8.

## RESULTS

The cases were reviewed based on demographic data, clinical details, tumour laterality, focality, gross and microscopic features along with IHC wherever performed. Out of 13 cases, four were diagnosed as papillary RCC and the remaining were oncocytic and chromophobe renal tumours.

### Papillary Renal Cell Carcinoma

The clinical and pathological data of the tumours diagnosed as papillary renal cell tumours are enumerated in Table 2.

**Table 2:** Clinical and pathological data of papillary RCC cases with revised diagnosis

**Case**	**Age/ Sex**	**Laterality**	**Gross appearance**	**Focality**	**Previous diagnosis**	**IHC**	**Final diagnosis**
1	63/M	Left	Single grey, brown tissue of 1.8cm	Unifocal	Papillary RCC type 2	PAX8/AMACR/CK7 +, TTF1-ve	Papillary RCC ISUP Grade 2
2	64/M	Left	Circumscribed	Unifocal	Cystic renal neoplasm -Clear cell RCC (Eosinophilic variant)/ oncocytoma	Not done	Papillary RCC ISUP Grade 2
3	54/M	Left	Circumscribed	Unifocal	Papillary RCC type 2 ISUP grade 4	AMACR/Vimentin+; CD117/CK7-ve	Papillary RCC ISUP Grade 4
4	32/F	Left	Infiltrative, friable, grey, white to brown	Unifocal	Papillary RCC type 2 ISUP grade 2	Not done	Papillary RCC ISUP Grade 3

The age distribution diagnosed as papillary renal cell carcinoma ranged between 32 and 64 years with a mean age of 53.25 years. The male to female ratio was 3:1. All tumours were unifocal and confined to the left kidney.

**Figure 1:**
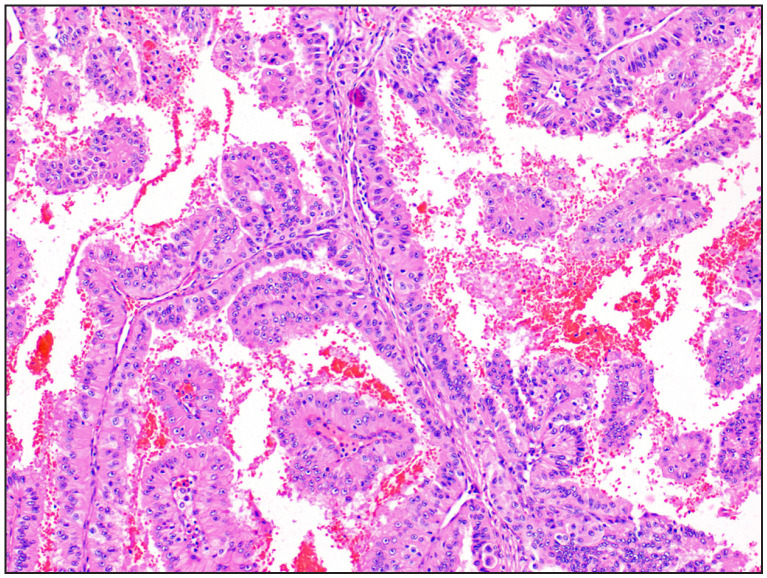
Case 4 Papillary renal cell carcinoma ISUP grade 3 was initially labelled as papillary renal cell carcinoma Type 2 ISUP grade 2 [H&E, 20x].

**Figure 2:**
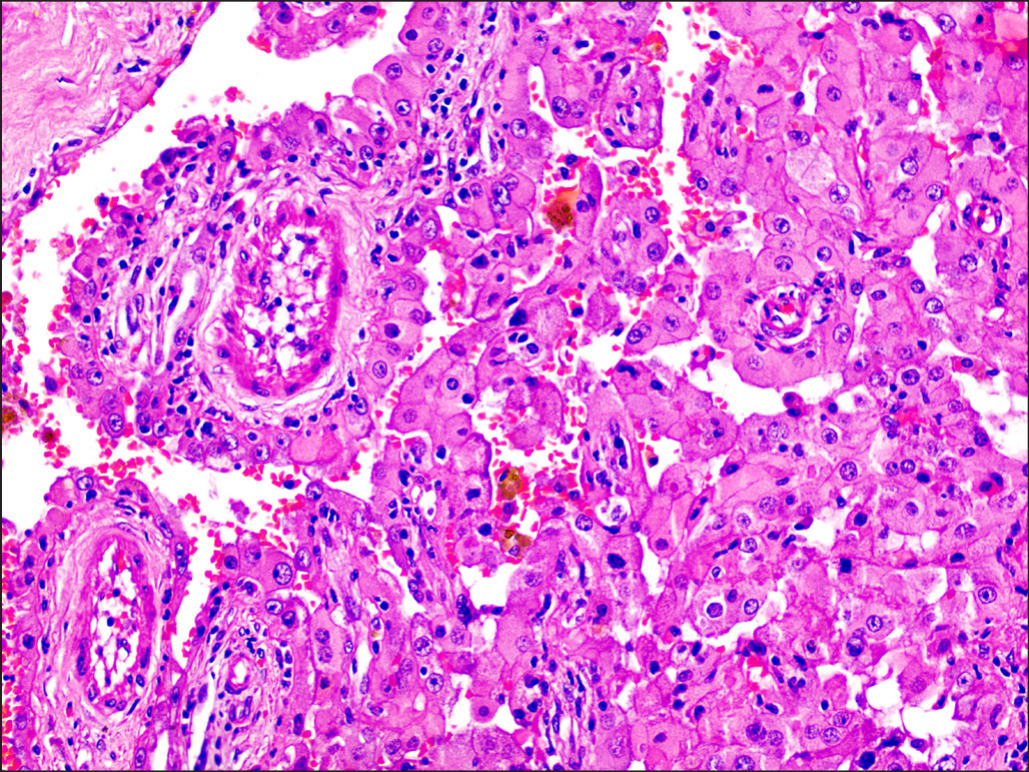
Case 1 Papillary renal cell carcinoma reclassified as ISUP grade 2 [H&E, 20x].

**Figure 3:**
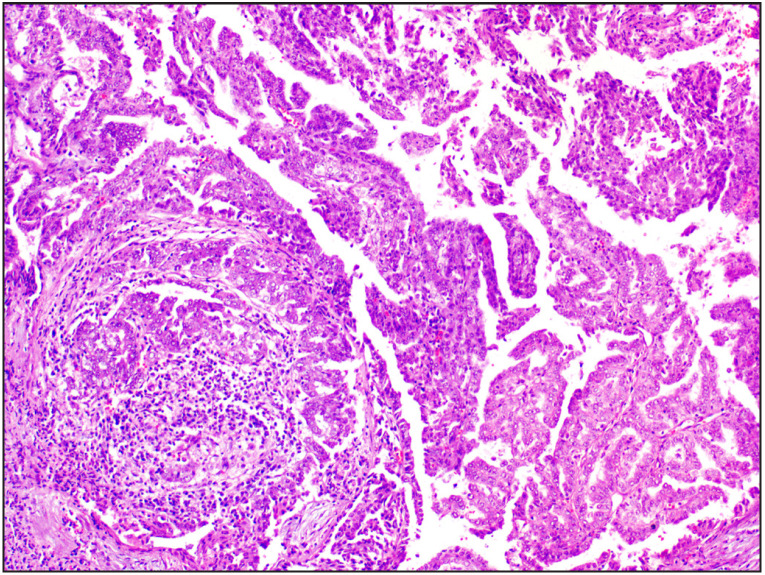
Case 2 Papillary renal cell carcinoma reclassified as ISUP grade 2 [H&E, 20x].

Out of four tumours, three cases that were diagnosed as papillary renal cell carcinoma type 2 based on previous WHO classification were reclassified to papillary renal cell carcinoma. Among these cases, the ISUP/WHO grade remain unchanged for case 3. However, the ISUP/WHO grade was changed from grade 2 to grade 3 in case 4 (Figure 1) and in case 1 (Figure 2) to grade 2, respectively. Case 2 (Figure 3), which was labelled as cystic renal neoplasm, was re-categorized and graded to papillary renal cell carcinoma ISUP grade 2 based on the morphological features.

Case 1 presented with metastases to liver, lung, ribs, vertebrae, and para-aortic lymph nodes. Morphologically, the tumour showed a tubulopapillary architecture. To confirm the primary origin of the tumour and tumour type, immunohistochemistry was performed. The tumour was positive for PAX8 and negative for TTF-1 indicating the renal origin of the tumour while positivity for AMACR & CK7 confirmed the diagnosis of papillary renal cell carcinoma.

### Oncocytic and Chromophobe Renal Tumours

Out of 13 cases, nine cases were oncocytic tumours. The age distribution for oncocytic tumours ranged between 39 and 72 years with a mean age of 53.22 years. The male to female ratio was 1:0.8. The majority of the tumours were in the left kidney and were unifocal except for one case which presented with metachronous renal cell carcinoma. The clinical and histopathological data are listed in Table 3.

**Table 3:** Clinical and pathological data of oncocytic tumour cases

**Case No**	**Age/ Sex**	**Laterality**	**Gross**	**Focality**	**Previous diagnosis**	**IHC**	**Final Diagnosis**
1	52/F	Left	Yellow, grey, brown,	Unifocal	Suggestive of Clear cell RCC ISUP grade 3	Not done	Chromophobe RCC
2	65/F	Left	Circumscribed, central scarring, brownish	Unifocal	Renal oncocytoma	CD117+; CD10/CK7-	Renal oncocytoma
3	59/F	Left	Variegated, yellow	Unifocal	Chromophobe RCC	CD117/E-cadherin+; Vimentin/S100/CK7-	Chromophobe RCC
4	42/F	Left	Exophytic, grey, white	Unifocal	Chromophobe RCC	CD117/CK7+; Vimentin-	Chromophobe RCC
5	72/M	Left	Central scar, mahogany brown	Unifocal	Renal oncocytoma	CD117+; CK7/Vimentin-	Renal oncocytoma
6	48/M	Right	Solid, tan, brown	Unifocal	Chromophobe RCC	CD117/CK7+	Chromophobe RCC
7	42/M	Left	Circumscribed, yellow, tan, central scar	Unifocal	Renal oncocytoma	CD117+; CK7-	Renal oncocytoma
8	60/M	Left	Multiple bits largest bit- 2.2x1.8x1.6cm	Multifocal	Oncocytic carcinoma of low malignant potential	CD117+; CK7 focal +; HMB45-	Renal oncocytoma
9	39/F	Right	Grey tan tumour	Unifocal	MiT family translocation RCC	CK+. AMACR/HMB45 -	Favouring TFEB-altered RCC requiring IHC confirmation

**Figure 4:**
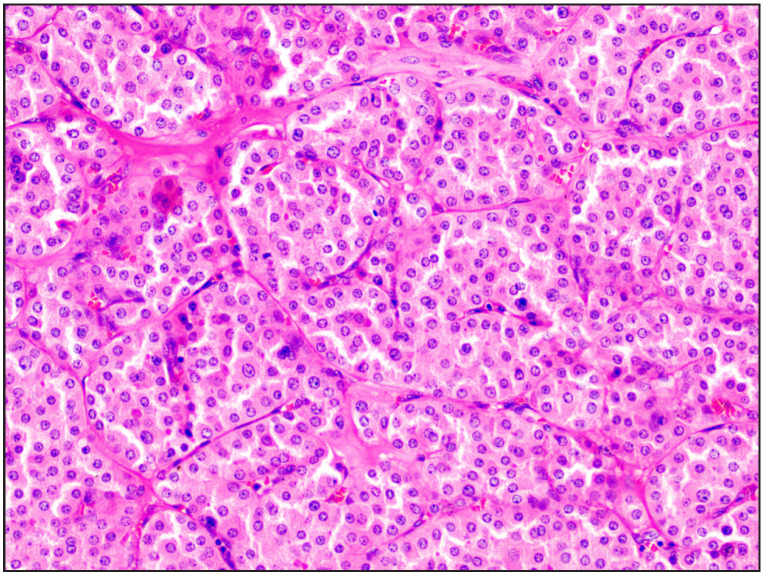
Case 8 Renal oncocytoma was initially labelled as oncocytic carcinoma of low malignant potential [H&E, 20x]

Case 8 (Figure 4) was reported as an oncocytic carcinoma of low malignant potential. Following review with the clinical details, histomorphology and immunohistochemistry, the diagnosis was changed to renal oncocytoma.

**Figure 5:**
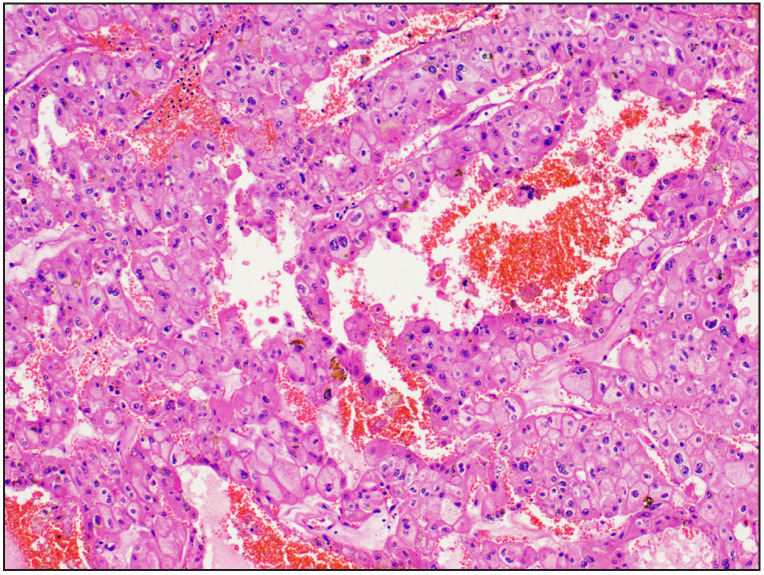
Case 1 Clear cell RCC ISUP grade 3 reclassified as Chromophobe renal cell carcinoma [H&E, 20x]

Out of four cases of chromophobe renal cell carcinoma, Case 1 (Figure 5) which was diagnosed as Clear Cell renal cell carcinoma ISUP grade 3, was reclassified as chromophobe renal cell carcinoma.

**Figure 6:**
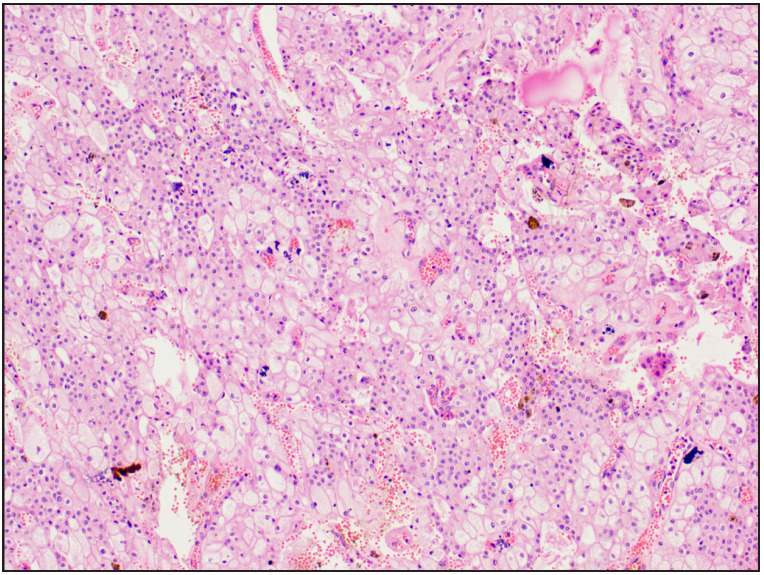
Case 9 Features suggestive of TFEB-altered renal cell carcinoma with dual cell population initially labelled as MiT family translocation renal cell carcinoma [H&E, 20x]

In Case 9 (Figure 6), a biphasic morphology was observed where one cell type had abundant voluminous cytoplasm surrounded by other small cell type with scanty eosinophilic cytoplasm. Available immunohistochemistry markers were not conclusive and further molecular tests were not done. The initial diagnosis rendered was MiT Family translocation RCC based on the morphology and reclassified as molecularly defined renal carcinomas with features suggestive of TFEB-altered renal carcinomas. Specific IHC markers were not available to confirm the diagnosis.

## DISCUSSION

The purpose of this study was to review renal tumours to reclassify papillary renal cell carcinoma, oncocytic and chromophobe renal tumours based on the 5th edition WHO classification, 2022 of urogenital tumours. The cases were re-evaluated with clinical details, microscopic findings and available immunohistochemical markers.

Papillary renal cell carcinomas account for 1-20% of all RCC and are no longer subtyped into type 1 and 2. Histologically, these tumours have a papillary architecture with a fibrovascular core, foamy histiocytes, and psammoma bodies. The tumours are positive for AMACR, CK7, Vimentin, and CD10 and need to be differentiated from papillary neoplasm with reverse polarity which are GATA3 positive, vimentin negative, and AMACR positive (5,6). These tumours have alterations in chromosomal numbers and are associated with mutations in MET, CDKN2A, SETD2, BAP1, PBRM1, NFE2L2, and mTOR genes and have a better prognosis when compared with clear cell RCC in the organ-confined stage (7).

In this study, out of the 4 cases of papillary renal cell carcinoma, Case 2 that was labelled as cystic renal neoplasm was re-categorised as papillary renal cell carcinoma and the grade of tumour for Case 4 and Case 1 was reassigned as Grade 2 and Grade 3 respectively. With discovery of future genetic alterations associated with papillary RCC, targeted therapies can be designed to improve survival outcomes for these molecular defined papillary tumour subtypes.

Oncocytoma and chromophobe renal cell carcinoma are classified and grouped under a single umbrella in the latest edition of the WHO classification of tumors. Chromophobe renal cell carcinoma show a characteristic morphology with distinct cell membrane of tumour cells with abundant clear to eosinophilic cytoplasm, perinuclear cytoplasmic clearing, and irregular wrinkled or raisinoid nuclei. These tumours are positive for CK7, CD117, Cathepsin K, and SDHB. Histologically, oncocytomas have round to polygonal cells with eosinophilic dense granular cytoplasm and round uniform nuclei with evenly distributed chromatin. Oncocytomas show immunopositivity for CD117, S100A, E-cadherin, pan-cytokeratin and CK7 is negative (4). In this study, one case of oncocytoma developed in a known case of bilateral clear cell renal cell carcinoma. This tumour had histological features of renal oncocytoma and expressed CD117 and focal CK7 positivity and was negative for HMB45. The final diagnosis of renal oncocytoma was confirmed by considering the multifocal, bilateral, and metachronous nature of the tumour. These features are documented in the literature and occur in 4 to 6% of the cases and coexistence of oncocytoma has been observed in 10% of cases (8).

Other oncocytic tumours defined by the recent 5th edition WHO classification are the Hybrid oncocytic chromophobe tumour (HOCT), Eosinophilic vacuolated tumour (EVT), and Low-grade oncocytic tumour (LOT). They can be solitary and sporadic. Multifocal and bilateral tumours are known to be associated with the Birt-Hogg-Dube syndrome where it shows the FLCN mutation and checkerboard mosaic pattern on morphology. EVT is associated with gene mutations involved in the mTOR pathway. Morphologically, the tumour cells have eosinophilic vacuolated cytoplasm and prominent nucleoli with entrapped tubules. Immunophenotypically, the tumours are CD117 positive and CK7 negative. LOT is also associated with gene mutations in the mTOR pathway and shows low grade nuclear features. In contrast to EVT, LOTs are CD 117 negative and CK7 positive (4,5).

Case 9 posed a diagnostic difficulty due to the biphasic nature of the tumour and inconclusive immunohistochemistry (CK+&AMACR/HMB45-). Additional molecular tests or immunohistochemistry for TFEB could not be performed and the patient was lost to follow up.

Odeh et al., re-evaluated renal cell carcinoma according to the 2022 WHO classification in a large cohort of 457 cases and found no discrepancy with the previous diagnoses (9). A recent study showed that two thirds of tumours which were “unclassifiable eosinophilic RCC” were reclassified based on the newer immunohistochemical markers and contributed to the prognostic outcomes (10). A single institute study on the recently introduced LOTs recommends using molecular testing for confirmation, larger cohorts, and longer follow up to characterize these tumours as a separate entity (11). An algorithm approach described by Amin et al.*,* is helpful to categorize low grade oncocytic neoplasms and provide adequate management strategies although newer defined tumours that require additional testing may remain underrecognised (12).

In low resource settings where these tests are not available, histomorphology continues to form the basis for diagnosis. Some tumours can be diagnosed with additional immunohistochemical markers and those that require molecular testing may be sent for additional testing to referral centers.

## CONCLUSION

Recent WHO classification has enumerated the new molecularly defined renal carcinomas that require advanced and expensive immunohistochemistry markers and molecular tests for confirmation of the diagnosis and targeted therapy. The diagnosis of these tumours may be challenging for the pathologists and hence many of the newer entities may remain undiagnosed without appropriate molecular testing.

## Limitations of the Study

This study employed the newly categorised renal tumours and recent modifications of the 5th edition of the WHO renal tumours to redesignate existing papillary, oncocytic, and chromophobe tumours. The limitation of the study was the small sample size and lack of specific IHC markers and molecular tests to confirm the new molecularly defined renal tumours.

## Funding

None.

## Conflict of Interest

The authors have no conflict of interest.
